# Retraction for: Circular RNA ZNF609 drives tumor progression by regulating the miR-138-5p/SIRT7 axis in melanoma

**DOI:** 10.18632/aging.205843

**Published:** 2024-05-15

**Authors:** Quan Liu, Wei Cui, Chao Yang, Li-Ping Du

**Affiliations:** 1 Department of Plastic Surgery, Sichuan Academy of Medical Sciences and Sichuan Provincial People’s Hospital, Chengdu 610072, Sichuan, China; 2 Department of Traditional Chinese Medicine Surgery, Sichuan Academy of Medical Sciences and Sichuan Provincial People’s Hospital, Chengdu 610072, Sichuan, China

**Keywords:** melanoma, progression, DNA damage, circZNF609, miR-138-5p, SIRT7

**This article has been retracted:** Aging has completed its investigation of this paper. We found that four images in **Figure 5F, 5G,** which present the results of comet assays in A375 (5F) and SK-MEL-28 (5G) cells treated with control mimic or miR-138-5p mimic, or co-treated with miR-138-5p mimic and pcDNA3.1-SIRT7, were also used in **Figure 3A, 3B**, where cells were treated with circZNF609 shRNA or control shRNA. The same images were used again in **Figure 4G, 4H,** which show the results of comet assays in A375 and SK-MEL-28 cells treated with control shRNA or circZNF609 shRNA, or co-treated with circZNF609 shRNA and miR-138-5p inhibitor. The authors informed the journal that a portion of the original data is missing, so it is impossible to correct the misused images. All of the authors agreed to retract the manuscript and signed the retraction agreement letter.

